# Strategies to Address Misestimation of Energy Intake Based on Self-Report Dietary Consumption in Examining Associations Between Dietary Patterns and Cancer Risk

**DOI:** 10.3390/nu11112614

**Published:** 2019-11-01

**Authors:** Nathan M. Solbak, Ala Al Rajabi, Alianu K. Akawung, Geraldine Lo Siou, Sharon I. Kirkpatrick, Paula J. Robson

**Affiliations:** 1 Cancer Research & Analytics, Alberta Health Services, 1820 Richmond Rd SW, Calgary, AB T2T 5C7, Canada; Nathan.Solbak@ucalgary.ca (N.M.S.); Ala.Rajabi@ahs.ca (A.A.R.); Losioug@gmail.com (G.L.S.); 2 School of Public Health and Health Systems, University of Waterloo; 200 University Avenue West, LHN 1713, Waterloo, ON N2L 3G1, Canada; Sharon.Kirkpatrick@uwaterloo.ca; 3 Cancer Research & Analytics and the Cancer Strategic Clinical Network, Alberta Health Services, Sun Life Place, 15th floor, 10123 99 Street NW, Edmonton, AB T5J 3C6, Canada; Paula.Robson@ahs.ca

**Keywords:** dietary patterns, energy misestimation, Alberta’s Tomorrow Project, revised Goldberg method, cancer incidence, diet-disease associations

## Abstract

The objective of this study was to determine the influence of strategies of handling misestimation of energy intake (EI) on observed associations between dietary patterns and cancer risk. Data from Alberta’s Tomorrow Project participants (*n* = 9,847 men and 16,241 women) were linked to the Alberta Cancer Registry. The revised-Goldberg method was used to characterize EI misestimation. Four strategies assessed the influence of EI misestimation: Retaining individuals with EI misestimation in the cluster analysis (Inclusion), excluding before (ExBefore) or after cluster analysis (ExAfter), or reassigning into ExBefore clusters using the nearest neighbor method (InclusionNN). Misestimation of EI affected approximately 50% of participants. Cluster analysis identified three patterns: Healthy, Meats/Pizza and Sweets/Dairy. Cox proportional hazard regression models assessed associations between the risk of cancer and dietary patterns. Among men, no significant associations (based on an often-used threshold of *p* < 0.05) between dietary patterns and cancer risk were observed. In women, significant associations were observed between the Sweets/Dairy and Meats/Pizza patterns and all cancer risk in the ExBefore (HR (95% CI): 1.28 (1.04–1.58)) and InclusionNN (HR (95% CI): 1.14 (1.00–1.30)), respectively. Thus, strategies to address misestimation of EI can influence associations between dietary patterns and disease outcomes. Identifying optimal approaches for addressing EI misestimation, for example, by leveraging biomarker-based studies could improve our ability to characterize diet-disease associations.

## 1. Introduction

Cancer continues to exert a large toll on morbidity and mortality globally [1]. Cancer prevention recommendations emphasize the importance of behaviors such as tobacco cessation, physical activity, and healthy eating [2]. With regard to characterizing healthy eating, there is a growing emphasis on moving beyond single dietary components to a more holistic approach that embraces overall eating patterns [3]. The relationship between diet and disease is complex: foods and beverages are consumed in different combinations that allow for countless interactions between nutrients and other dietary components such as phytochemicals, making attributing health effects to a single dietary component difficult [4]. Examining dietary patterns and their associations with cancer risk acknowledges this complexity and could lead to improved estimates of diet-cancer associations [5], as well as clearer recommendations for promoting health and reducing disease risk.

Epidemiological studies investigating associations between eating patterns and disease risk are typically reliant on self-reported intake captured using tools such as food frequency questionnaires (FFQ) [6]. However, all self-report dietary intake data are characterized by measurement error, exhibited by differences between observed and true intake values [7]. Multiple factors contribute to measurement error, including imperfect recall of intake over long time periods (leading, for example, to omission of consumed foods or beverages or inaccurate portion size estimates), social desirability biases, and characteristics of the tools themselves, such as incomplete food lists and portion size options within FFQs [8]. Measurement error can obscure associations that truly exist, leading to inconsistencies in estimated associations between eating patterns and disease risk [9].

Evidence from validation studies indicates that estimation of energy intake (EI) is particularly affected by measurement error [10]. Given that almost all foods and beverages contribute energy, even small errors in reporting of individual foods and beverages can compound to result in energy misestimation [7]. Misestimation of EI occurs when there is a discrepancy between reported EI and energy expenditure (EE), assuming that an individual has a relatively stable body weight [11]. The optimal method for measuring EE is the doubly labeled water (DLW) technique. However, DLW—a recovery marker able to provide unbiased estimates of true EI—can usually be administered only in small samples due to the cost and participant burden [12]. Thus, while DLW is very useful for validation and calibration studies, it is not feasible for large-scale studies. Nonetheless, its use in biomarker-based validation studies has provided evidence that the difference between true and reported EI among adults based on FFQs may be substantial (in the range of 28%) [13] and larger than that observed for other dietary components for which biomarkers are available, including protein and potassium [13,14].

For the purpose of population-based research, alternative methods have been developed to assess the plausibility of reported EI derived from self-reported food and beverage consumption in relation to EE based on basal metabolic rate (BMR) and physical activity level (PAL) [11]. For example, the revised Goldberg method [15] uses an equation to predict total EE [16,17]. Assuming that changes in body weight can be ignored at the group level, observed EI should equal total EE [15]. Cut-offs can then be used to classify participants based on the plausibility of their EI compared to their estimated EE. Tooze et al. [18] reported that, compared to DLW, the revised Goldberg method had a sensitivity of >92% for identifying participants whose EI estimates were affected by misreporting based on FFQ data.

Once energy misestimation is characterized, researchers must determine how to handle it in their analyses. Excluding individuals determined to have implausible EI estimates is not recommended but it has been suggested that analyses are stratified based on energy reporting status [19] or that the EI:EE ratio be included in statistical models to account for energy misestimation [20,21,22]. There has been relatively little attention to the impact of strategies for addressing EI misestimation in analyses seeking to examine associations between dietary patterns and disease risk [3]. Thus the objective of this study was to determine the influence of different strategies for addressing EI misestimation on observed associations between dietary patterns, determined using *k*-means clustering, and risk of all cancers, a subgroup of cancers with strong evidence of association to diet, and digestive system cancers, among adults.

## 2. Materials and Methods

### 2.1. Data Source

Alberta’s Tomorrow Project (ATP) is a prospective cohort of ~55,000 Albertans established in 2000 to facilitate studies into the etiology of cancer and chronic diseases. Recruitment, enrollment, and data collection methods are described in detail elsewhere [23,24,25]. Briefly, Albertans aged 35–69 years at enrollment, with no history of cancer except non-melanoma skin cancer, were recruited by telephone-based random digit dialing which facilitated balanced recruitment across the province. Eligible participants were mailed a consent form and a Health and Lifestyle Questionnaire (HLQ), followed by a past-year FFQ (Canadian Diet History Questionnaire-I; CDHQ-I), and the Past-Year Total Physical Activity Questionnaire (PYTPAQ). Participants had the opportunity to consent to linkage with the Alberta Cancer Registry (ACR) and provided personal health numbers. All questionnaires were sent via postal mail to participants who returned completed questionnaires in pre-paid envelopes.

Inclusion in the current study was limited to participants who consented to administrative data linkage and completed the HLQ, PYTPAQ, and CDHQ-I. Participants were excluded if they resided outside of Alberta (*n* = 29), had a prior cancer diagnosis, except for non-melanoma skin cancer, assessed via ACR linkage (*n* = 71), were recruited as the second ATP member in their household (*n* = 342) (due to potential intra-class correlations among members of the same household), were pregnant (*n* = 63), or were characterized as underweight (body mass index (BMI) <18.5) based on self-reported heights and weights (*n* = 18) (due to potential association between underweight and increased risk of disease [26]). Additionally, participants with missing height or weight measures (*n* = 70) were excluded since these values are required to calculate BMR for the purpose of the revised Goldberg method. The final sample sizes were *n* = 9,847 men and *n* = 16,241 women.

### 2.2. Dietary Intake Assessment

The CDHQ-I is a 257-item past-year FFQ based on the Diet History Questionnaire developed by the U.S. National Cancer Institute [27] and modified to reflect food availability, brand names, nutrition composition and food fortification in Canada [28,29]. Responses to the CDHQ-I were analyzed using Diet Calc software (version 1.4.2; National Cancer Institute, MD, USA) and a nutrient database tailored to the CDHQ-I, resulting in data on intake of energy, 66 nutrients, and 284 single foods. On the basis of similarities in macronutrient composition and culinary use, the 284 single foods were categorized into 55 food groups [30]. The percentage of daily total EI contributed by each of the 55 food groups was calculated by dividing daily EI provided by each food group by daily total EI.

### 2.3. Physical Activity Assessment

The PYTPAQ collects domain-specific (transportation, occupational, household and recreational) information on frequency, duration, and intensity of physical activity in the past 12 months [31]. The PYTPAQ has been evaluated relative to accelerometer data, showing acceptable reliability (0.64) and validity (0.41) for measurement of past-year physical activity [31].

### 2.4. Energy Intake Estimation

For EI estimation, participants were classified as EI under-reporters, plausible-reporters, or over-reporters using the revised Goldberg method [15,32]. Briefly, the plausibility of total reported energy intake (rEI) was determined based on the 95% confidence limits of agreement (cut-offs) between the ratio of total rEI to BMR and the ratio of total EE to BMR (PAL). BMR was calculated based on the participant’s age, sex, body weight, and standing height using the Mifflin equation [33]. EE was calculated based on BMR, physical activity (sum of all domains from the PYTPAQ), and body weight [34]. To account for skewness in the distribution of rEI, the rEI to BMR ratio was transformed to a logarithmic scale. Individuals with rEI:BMR to PAL values below the lower Goldberg cut off, above the upper Goldberg cut off, and within Goldberg cut-offs were identified as under-reporters, over-reporters, and plausible-reporters, respectively. The Goldberg cut-offs were: lower = 0.75270, upper = 2.07586 for sedentary, lower = 0.90324, upper = 2.49103 for low active, lower = 1.05378, upper = 2.90620 for active and lower = 1.32475, upper = 3.65351 for very active.

### 2.5. Cancer Incidence and Sub-Groups

Primary incident cancer cases (All-Cancers, except non-melanoma skin cancer) were obtained by linkage to ACR in July 2017. The International Classification of Diseases for Oncology 3rd edition (ICD-O-3) was used to identify individual cancers. A subgroup of 21 primary cancers were identified based on a matrix from the World Cancer Research Fund/American Institute for Cancer Research Continuous Update Project (WCRF/AICR CUP) reporting on dietary components with convincing or probable evidence for increased or decreased risk of cancer [35] (Dietary-Cancers; Table 1**)**. Another subgroup of 11 primary cancers were chosen based on the World Health Organization (WHO) classification of digestive system cancers [36] (Digestive-Cancers; Table 1).

Follow-up time was calculated from the age at enrollment to the age at cancer diagnosis or at ACR linkage for participants who remained cancer-free during the follow-up period. All age variables were expressed with up to 2 decimal places for precision. To account for competing risk during follow-up due to death in participants who were cancer-free, vital statistics data were obtained from Alberta Health Services Data Integration, Measurement and Reporting (DIMR). In participants who remained cancer-free but died before linkage to ACR, follow-up time was calculated from age at enrollment to age at death.

Linkage with the ACR identified 2276 primary cancer cases (All-Cancers; 982 men and 1294 women) over 33,6524.5 person-years follow-up (median (IQR)= 13.1 (5.0) years). For Dietary-Cancers and Digestive-Cancers, there were 1169 (264 men and 905 women) and 392 cases (191 men and 204 women), respectively.

### 2.6. Statistical Analysis

**Figure 1:**
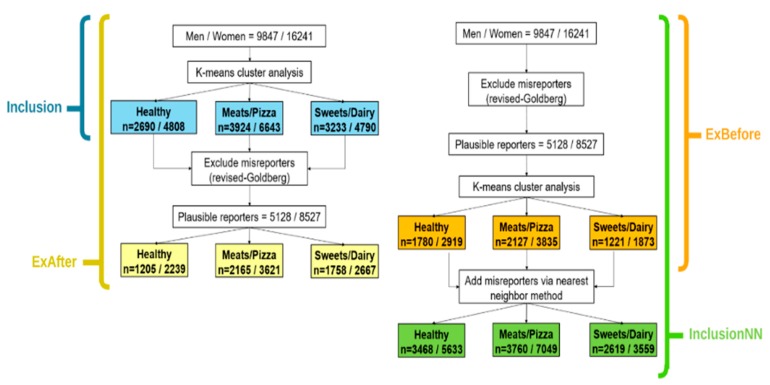
Flow chart illustrating different methods for accounting for potential misreporting of energy intake.

*k*-means cluster analyses [37] were performed to characterize dietary patterns. Individuals whose EI was determined to be affected by misestimation (EI under-reporters and over-reporters, henceforth collectively grouped as EI misreporters because over—reporters comprised only 1% of the study sample—and could not be assessed separately) were accounted for in the cluster analyses using four methods: included in the cluster analysis (Inclusion); excluded prior to completing the cluster analysis (ExBefore); excluded after completing the cluster analysis (ExAfter); and finally, excluded before the cluster analysis but added to the ExBefore cluster solution using the nearest neighbour method (Inclusion-NN) [38] (Figure 1). The nearest neighbour method (*k* = 1) is a pattern classification method that measures the Euclidean distance between a test example (i.e., participant) and the data set and assigns the test example to the cluster of the nearest neighbour [38].

All analyses were stratified by sex as self-reported by participants. The percentages of total rEI contributed by each of the 55 food groups were used as input variables. The k-means cluster analyses method started with the researcher selecting *k* initial clusters (a positive integer representing the number of clusters) and initial cluster seeds (a random positive integer representing the initial number of participants to be assigned to each cluster). Subsequently, each additional participant was automatically assigned to the nearest cluster on the basis of Euclidean distance, forming temporary clusters. Seeds were then replaced by the centroid of each temporary cluster, with the “centroid” referring to the mean observation of a cluster. Each participant was then reassigned to the nearest centroid, updating the location of the centroids. The process was repeated until centroids did not significantly change location. For these analyses, between two and seven cluster solutions were tested to balance feasibility and robustness. To reduce the impact of local optima [39], cluster analyses were run 10 times with different random starting seeds for each cluster solution. In both men and women, the cluster solution that provided the minimum total within-cluster sum of squares distance was selected. For all selected cluster solutions (2 to 7), the between- and within-cluster variances for each food group were calculated. Then, the natural log-transformed ratios of the between- versus within-cluster variances were calculated to compare heterogeneity between and within clusters. The further apart the clusters, the larger the ratio; therefore, the optimal number of clusters is given by the cluster solution that has many food groups with large ratios. Dietary patterns were established by including each food group in the cluster to which it contributed the highest rEI. As such, food groups included in each of the three dietary patterns are mutually exclusive.

Before cluster analysis, each input variable was standardized by subtracting the minimum input value and then dividing by the range. This standardization method, known as the range method, has been reported to give consistently better recovery of cluster structure in different error conditions, separation distances, clustering methods, and coverage levels when compared with other standardization methods, such as the *z* score [40].

Multivariable Cox proportional hazard regression models were used to assess the associations between observed dietary patterns and cancer risk, including All-Cancers, Dietary-Cancers, and Digestive-Cancers. Adjusted hazard ratios (AHR) were estimated in comparison to the association of a reference pattern with cancer outcomes. Competing risk analysis was performed, with the standard multivariable Cox proportional hazard regression model applied to the cause-specific hazard of interest and competing events treated as censored observations [41]. Regression models were adjusted for age (modelled on a continuous scale), BMI (modelled on a continuous scale), leisure-time physical activity (MET hours/week; modelled on a continuous scale), marital status, educational attainment, smoking status, family history of cancer, and personal history of chronic disease. In models for women only, menopausal status and hormone replacement therapy usage were included.

Means and SD are presented for continuous variables, and counts and percentages for categorical variables. For interpretation purposes, comparisons examined whether associations would be considered significant based on the often used *p*-value threshold of <0.05, though the consistency of estimates across methods of accounting for EI misestimation is also considered more holistically given that this p-value threshold is arbitrary [42]. All analyses were performed using SAS statistical software (version 9.2-Linux, SAS Institute, INC., Cary, North Carolina, USA).

## 3. Results

### 3.1. Participant Baseline Sociodemographic Characteristics

Three dietary patterns, or clusters, were identified for both men and women: Healthy, Meats/Pizza, and Sweets/Dairy. Baseline sociodemographic characteristics stratified by dietary pattern and EI reporting status are presented in Table 2. Higher proportions of men and women assigned to the Meats/Pizza pattern were affected by obesity (BMI ≥ 30), while lower proportions had BMI < 25, compared to participants in both the Healthy and Sweets/Dairy patterns. Men and women in the Healthy pattern had higher reported leisure-time physical activity values compared to their counterparts in the Sweets/Dairy and Meats/Pizza patterns. The highest proportions of current smokers for both men and women were in the Meats/Pizza pattern. In men, the proportion who reported a personal history of chronic disease was highest in the Healthy pattern while in women, the proportion who reported a personal history of chronic disease was very similar across dietary patterns. For both men and women and across all dietary patterns, higher proportions of misreporters were affected by obesity, while lower proportions had BMI < 25, compared to plausible reporters. The proportions of EI misreporters were very similar between men (47.9%) and women (46.8%) and across all cancer cases and non-cases in men (49.0% vs. 47.8%) and in women (48.6% vs. 46.7%). 

### 3.2. Dietary Patterns in Relation to Methods for Accounting for Misestimation of Energy Intake 

The greatest contributors to total rEI in each dietary pattern across different methods of accounting for EI misreporters is summarized in Table 3. With few exceptions, the majority of the food groups in all three dietary patterns were common across the different methods in both men and women. However, Other Breads was not included in the Meats/Pizza pattern within the ExBefore and InclusionNN methods among men and ExBefore method in women. The percentage contribution of food groups in all three dietary patterns were very similar across different methods of accounting for EI misreporting. For the Inclusion method, fruits, high-fiber breakfast cereal, fruit juices, rice and nuts contributed the greatest proportions of energy for men within the Healthy pattern. For women in the Healthy pattern under the Inclusion method, fruit, regular-fat dairy products, lean fat poultry, nuts and rice were the largest contributors to total EI. Men assigned to the Meats/Pizza pattern with Inclusion had the highest total rEI contribution from meats, pasta/pizza, beer, regular soda and chips; while women in the Meats/Pizza pattern had similar intakes except for beer. Men and women assigned to the Sweets/Dairy pattern with Inclusion had high total rEI of low-fat dairy products and wholemeal (whole-grain) bread, and several sweets such as cakes, jams and ice cream. Mean intakes of plausible reporters in the ExBefore and ExAfter methods were similar in both men and women. Women in the Sweets/Dairy pattern ExBefore had only 3 food groups with the highest percentage contribution of total rEI compared to 7 food groups in the ExAfter. The largest contributors of rEI were similar between ExBefore and InclusionNN in both men and women. The mean intake of some food groups varied across different methods for accounting for potential misreporting of EI, this changed the ranking of food groups but the overall dietary patterns remained the same.

### 3.3. Association between Dietary Patterns and Cancer Risk

For All-Cancers, no significant associations were observed between dietary patterns and cancer risk in men, regardless of the method used to account for misestimation of EI. However, the point estimates for the Sweets/Dairy and Meats/Pizza patterns and All-Cancer were higher in the ExAfter and Inclusion methods, respectively, compared to the other methods of accounting for EI misreporting. In women, a significant increased cancer risk was associated with the Meats/Pizza pattern in the InclusionNN (AHR (95%CI): 1.14 (1.00–1.30)) method and in the Sweets/Dairy pattern for the ExBefore (AHR (95%CI): 1.28 (1.04–1.58)) method (Table 4). Among women, the point estimate for the Meats/Pizza pattern under the Inclusion method was very similar to the estimate for InclusionNN, but the former would not be considered a statistically significant association if applying a *p*-value threshold of <0.05.

For Dietary-Cancers, the Meats/Pizza pattern was associated with increased cancer risk among men under both the Inclusion (AHR (95%CI): 1.42 (1.00–2.02)) and ExAfter (AHR (95%CI): 1.92 (1.12–3.29)) methods (Table 5). Also among men, the Sweets/Dairy pattern was associated with increased cancer risk under the InclusionNN (AHR (95%CI): 1.45 (1.07–1.97)) and ExBefore (AHR (95%CI): 1.74 (1.12–2.72)) methods (Table 5). In women, no significant associations were observed for this subset of cancers (Table 5).

For Digestive-Cancers, no significant associations were observed with dietary patterns among men. Among women, a significantly increased risk of digestive cancers was observed for the Meats/Pizza pattern under the InclusionNN method (AHR (95%CI): 1.43 (1.02–2.01)) and the Sweets/Dairy pattern under the ExBefore method (AHR (95%CI): 1.73 (1.03–2.89)) (Table 6).

Competing risk analysis to account for deaths before ACR linkage date in participants who were cancer-free during follow-up did not significantly change the observed hazard ratios (Supplementary Materials: Tables S1–S3).

## 4. Discussion

The findings of this study suggest that misestimation of EI, ascertained using a prediction equation and self-reported physical activity and body weight and height, was prevalent among adults whose dietary intake was characterized using a FFQ within the context of a cohort study. Further, differing methods to account for this misestimation appear to impact observed associations between dietary patterns and cancer risk. Among men, there were no significant associations between dietary patterns and risk of all cancers regardless of the method of handling EI misestimation. However, the point estimates for All-cancers risk associated with the Sweets/Dairy and Meats/Pizza patterns were higher in ExAfter and Inclusion methods, respectively, compared to the other methods of accounting for EI misreporting. Among women, the Meats/Pizza pattern was associated with a 14% increased risk of all cancers in the method that included all participants regardless of EI misestimation (similar to that observed in the InclusionNN method). The Sweets/Dairy pattern was associated with a 28% increased risk of all cancers in the method that excluded women whose EI estimates were deemed to be affected by misestimation following the cluster analyses. Similarly, associations between dietary patterns and risk differed based on how EI misestimation was addressed for the subgroup of primary cancers for which there is evidence of the influence of dietary risk factors (men and women) and for digestive cancers (women). However, given that there is no marker of true dietary patterns, it is not possible to ascertain which method for accounting for EI misestimation results in observed associations that are the closest to truth.

Other studies have similarly suggested that analytical approaches used to account for potential EI misestimation can impact observed associations between dietary intake and disease outcomes among adults. A cross-sectional study of Norwegian women aged 50–69 years [44], which used an FFQ, found that self-reported CVD was significantly positively associated with “Western” dietary pattern scores among plausible reporters but not among all reporters. A prospective cohort study of Swedish adults [45] which used an interview-based diet history method, reported an increased risk of breast cancer with high alcohol intakes, with stronger risk estimates among plausible reporters compared with all reporters. A prospective cohort study of US adults [46] investigated the effect on the association between risk of breast, colon, endometrial and kidney cancer with reported EI calibrated to DLW data. Calibrated energy consumption was positively associated with risk of breast, colon, endometrial and kidney cancer, while uncalibrated energy was not. However, these studies reported lower proportions of misreporters (e.g., Norwegian 18%, Swedish 18% in men and 12% in women) compared to the current study (50% in both men and women). This could be due to the different equations used for calculating BMR. In the current study, BMR was calculated using the Mifflin equation while the Schofield and the Oxford equations were used in the Norwegian and Swedish studies respectively. In a study conducted with Korean adults [47], energy under-estimation was estimated to affect 14% of men and 23% of women, lower than the proportions observed in this study. This may be attributed to the use of a 24-hour recall in the Korean study as opposed to an FFQ in the current study.

Despite slight differences in methodology and design, the findings of this study are in line with previously published results indicating that estimated diet-disease associations can be influenced by measurement error [44]. Associations between dietary patterns and cancer risk varied depending on the methods used to account for misestimation of EI. Importantly, comparisons of findings based on different methods within and between studies are affected by considerations of what constitutes significant differences. For example, for women, the hazards ratios for cancer associated with the Meats/Pizza pattern were almost identical under two methods of accounting for energy misestimation, but under the conventional practice of applying a threshold of *p* < 0.05, only one of the two would be interpreted as significant. Thus, the findings highlight the need to consider not only how EI misestimation is accounted for across studies, but also to improve the reporting and interpretation of findings within nutritional epidemiology [42].

Prior analyses have highlighted the importance of considering measurement error and identified the need for caution in terms of the interpretation of diet-disease associations that have not been, at least partially, corrected for this error [45,46,48]. For example, regression calibration approaches are well developed and can make use of reference data, such as those collected using biomarkers or a less-biased tool such as 24-hour recalls in a subsample, to somewhat mitigate the impact of measurement error on diet-disease associations in large cohort studies in which an FFQ is the main tool [49]. Given that data from recalls have been shown to be affected by systematic measurement error to a lesser extent than data from FFQ [13], cohort studies administering recalls as the main assessment tool may be helpful for advancing our understanding of dietary intake and health. This is particularly true in the context of patterns since recalls provide comprehensive data including details on eating occasions and foods and beverages consumed in combination [7]. The use of recalls in cohort studies has become increasingly feasible with technological advances, such as online and mobile device-based tools [50]. Using such tools, cohort studies of the future can potentially take advantage of multiple modes of dietary assessment to dampen measurement error and its implications for observed diet-disease associations [51].

However, many current sources of data on diet and disease outcomes, with sufficient time elapsed from baseline data collection for cases of cancer and other conditions to accrue, may not provide opportunities for regression calibration. In this study, no reference data are available and we opted to use the revised Goldberg method to attempt to account for measurement error exhibited as EI misestimation. However, this method has challenges. The use of EI/BMR for evaluating EI depends on knowledge of energy requirements or EE [15]. For the purposes of the calculations, self-reported physical activity and anthropometric data were used—these data also undoubtedly contain measurement error, potentially resulting in misclassification of individuals based on their energy reporting status. Furthermore, the Goldberg method pertains to misestimation of energy only. It is known that misreporting is differential among different types of foods, beverages, and dietary components. For example, based on recovery biomarker-based studies, protein and potassium are less affected by misestimation than is energy [13]. This may be because errors in EI accumulate over many foods and beverages [7] but it may also be because energy-dense items are less accurately reported than other foods due to social desirability biases [52]. Studies based on observation and weighing have shown that different types of foods and beverages may be reported with differing levels of accuracy [53]. This is particularly relevant to studies of dietary patterns given that interest is inherently in combinations of foods and beverages consumed and the implications for health and disease risk.

Several statistical techniques are available for identifying dietary patterns and the choice of method depends largely on the research question at hand [54]. For example, cluster analysis may be useful for identifying mutually exclusive groups which differ according to their reported diet [54,55,56] and, as such, may help identify those at greater risk for developing specific cancers [57] or other chronic diseases. Alternatively, cluster analysis may group together those who tend to misreport their food and beverage consumption in similar ways, for example, due to social desirability biases. In this study, 55 food groups were created from the original 284 items in the FFQ while other studies have used smaller [54,55,56,58,59,60,61] or larger [57,62,63,64] numbers of food groups, potentially influencing the findings. The k-means method has limitations, including the need to pre-specify the number of clusters to retain, sensitivity to initial cluster seeds [65], and challenges posed by the existence of clusters of different size or shapes or those that may be nonspherical or occur across several subspaces [66]. Other studies have used principal component analysis [44,67,68,69], which aggregates food groups in linear combinations called principal components according to the extent to which they are correlated with each other. Studies using both k-means clustering and principal components analysis have observed similar patterns to those observed here. For example, Maree et al. [70] reported three dietary patterns in Australian men and women using k-means cluster analysis, with two of the clusters similar to the Healthy and Meats/Pizza patterns observed in the current study. Also using k-means cluster analysis, Freitas-Vilela et al. [71] also reported three dietary patterns, labelled Fruits and Vegetables, Meat and Potatoes and White Bread and Coffee, among pregnant women. Despite differences in naming, the three patterns are similar to those observed here. Further, using principal components analysis, Markussen et al. [44] identified similar patterns, named Prudent, Western and Continental, among both plausible and all reporters in a sample of women aged 50–60 years. Repeatability of dietary pattern analysis is often critiqued, since each cohort study can produce different patterns due to large variation between studies and their participants. However, the use of principal component analysis or cluster analysis appears to result in somewhat similar named dietary patterns.

This study made use of an existing cohort with a large sample size and careful validation of data and few missing values [24]. One exception was household income, which was characterized by a high degree of missingness and was not included in the Cox regression analysis despite evidence that socioeconomic status is associated with several types of cancers [72,73]. Cancer outcomes were ascertained via linkage with an accredited cancer registry (ACR), providing a more accurate diagnosis of the disease compared to self-report [44]. However, for privacy reasons, ATP does not release exact date of events and age at cancer diagnosis, given up to two decimal places, was used as an approximation for date of event. Thus, for the Cox regression analysis, precise follow-up times could not be calculated and therefore, hazard ratios might not have been precisely estimated. Due to the arbitrary nature of cluster analysis used in this study, the assignment of dietary patterns for an individual participant could have been different across methods of accounting for EI misreporting. This could also explain why differing methods of accounting for misestimation appear to impact observed associations between dietary patterns and cancer risk. For the k-means cluster analysis, total rEI was chosen as the input variable because EI is the foundation of the diet. All other nutrients must be provided within the quantity of food consumed to fulfill energy requirements. Therefore, if total EI is misreported, other dietary components may also be mis-estimated, albeit to differing degrees [74]. Other studies have used different measures such as the daily intake frequencies [70] and the average weight of food consumed per day [75]. These different measures may impact the results of the cluster analysis and hence the estimated diet-disease association. Finally, in addition to measurement error affecting the FFQ data, other variables, including physical activity, heights, and weights, are also subject to reporting error, potentially impacting the characterization of energy misestimation and the observed associations [76].

## 5. Conclusions

The results of this study suggest that observed associations between dietary patterns and health outcomes vary in relation to strategies for addressing EI misestimation. It is possible cohort studies that include the administration of biomarkers such as DLW in a subset of participants can shed light on misreporting of different dietary components and optimal strategies for accounting for it. Advances are also needed to enable improved characterization of dietary patterns, which inherently involve intake of many different foods and beverages that may be reported with different levels of accuracy. In the meantime, researchers should carefully consider how misestimation and other sources and symptoms of measurement error are characterized and accounted for and carefully report these details to enable appropriate interpretation of their findings.

## Figures and Tables

**Table 1:** Summary of primary cancers used in subgroup survival analyses.

Cancer location	ICD Code	Morphology Code ^c^
Dietary-Cancers ^a^		
Mouth	C1-C6, C9	
Pharynx	C10, C11, C13	
Larynx	C32	
Esophagus-squamous cell carcinoma	C15	Include only 8051, 8070, 8074, 8083
Lung	C34	
Stomach	C16	
Liver	C22	
Colon	C18, C26.0	
Rectosigmoid and rectum	C19, C20	
Breast	C50	
Endometrium	C54.1	
Kidney	C64	
Digestive-Cancers ^b^		
Esophagus	C15	
Stomach	C16	
Small Intestine	C17	
Colon	C18, C26.0	
Rectosigmoid and rectum	C19, C20	
Anus, anal canal and anorectum	C21	
Liver and intrahepatic bile ducts	C22	
Gall bladder and extrahepatic bile ducts	C23-24	
Exocrine pancreas	C25	Include only 8500, 8480, 8490, 8560, 8020, 8035,8154, 8441, 8470,8453, 8550, 8551, 8154, 8971, 8452

^a^ Diet-related cancers based on World Cancer Research Fund/American Institute for Cancer Research Continuous Update Project; ^b^ Digestive system cancers based on World Health Organization classification; ^c^ For both Dietary-Cancers and Digestive-Cancers, cases excluded morphology codes 9050–9055, 9140, 9590–9992.

**Table 2:** Baseline sociodemographic characteristics by EI reporting status and dietary pattern.

Reporting Status	Dietary Pattern								
	Healthy			Sweets & Dairy			Meats & Pizza		
	Total	Plausible Reporters	Misreporters	Total	Plausible Reporters	Misreporters	Total	Plausible Reporters	Misreporters
**Men**									
	*n* = 2690	*n* = 1205	*n* = 1485	*n* = 3233	*n* = 1758	*n* = 1475	*n* = 3924	*n* = 2165	*n* = 1759
Age at enrollment, median (IQR)	52.0 (15.3)	51.5 (15.9)	52.3 (14.8)	52.4 (15.6)	52.3 (16.1)	52.6 (15.0)	48.3 (12.4)	48.2 (12.9)	48.3 (12.1)
Body mass index^b^, %									
<25.0	26.7	33.6	21.0	25.3	30.3	19.5	18.8	22.9	13.8
25.0–29.9	49.6	49.3	49.8	50.1	49.5	50.8	48.2	49.6	46.6
≥30.0	23.8	17.1	29.2	24.6	20.3	29.8	33.0	27.5	39.7
Leisure-time physical activity(MET hrs/week) median (IQR)	27.5 (36.6)	26.5 (34.1)	28.6 (38.4)	17.9 (28.2)	18.0 (26.9)	17.8 (19.7)	18.0 (28.8)	17.8 (28.8)	18.2 (29.0)
Marital status, %									
Married/with partner	82.8	83.8	81.9	84.0	85.1	82.7	82.7	82.4	83.1
Single	7.1	7.1	7.1	5.9	6.4	5.3	6.4	6.5	6.2
Divorced/separated/widowed	10.1	9.1	11.0	10.1	8.5	11.9	10.9	11.1	10.7
Education, %									
Post-secondary complete	66.0	69.5	63.2	54.7	57.5	51.4	51.9	54.0	49.2
Some post-secondary	17.9	16.2	19.3	17.9	16.1	19.9	19.0	17.4	20.9
High school complete	8.9	7.6	10.0	14.9	14.6	15.3	18.4	16.8	20.2
High school not complete	7.2	6.8	7.5	12.6	11.8	13.4	10.8	11.7	9.7
Annual household income, %									
<$50,000	20.9	21.2	20.5	29.5	29.9	29.0	21.3	22.7	19.6
$50,000–$99,999	42.0	41.0	42.8	44.4	44.8	43.9	45.7	44.7	47.0
≥$100,000	36.0	36.2	35.8	24.5	23.7	25.4	31.6	31.1	32.3
Smoking status, %									
Never smoked	51.2	52.1	50.5	41.6	41.0	42.3	36.1	35.6	36.7
Former smoker	41.6	40.9	42.2	40.9	40.4	41.5	37.9	36.3	39.8
Current smoker	7.1	7.0	7.3	17.5	18.5	16.2	25.9	28.0	23.3
Family history of cancer, %									
No	50.2	50.0	50.0	47.9	40.0	47.8	51.1	51.3	50.9
Yes	49.9	50.0	50.0	52.1	60.0	52.2	48.9	48.7	49.1
Personal history of chronic disease ^a^, %									
None	48.8	50.9	47.1	52.5	52.3	52.8	54.5	56.7	51.7
One	29.5	28.4	30.4	28.6	28.9	28.3	28.7	27.7	29.9
Two or more	21.6	20.8	22.4	18.8	18.8	18.9	16.8	15.6	18.3
**Women**									
	*n* = 4808	*n* = 2239	*n* = 2469	*n* = 4790	*n* = 2667	*n* = 2123	*n* = 6643	*n* = 3621	*n* = 3022
Age at enrollment, median (IQR)	51.9 (14.0)	52.6 (14.2)	51.3 (13.8)	51.9 (16.0)	52.4 (16.7)	51.6 (15.2)	47.6 (13.4)	47.8 (13.4)	47.5 (13.3)
Body mass index ^b^, %									
<25.0	43.4	51.1	36.2	42.7	49.9	33.6	35.7	41.1	29.3
25.0–29.9	34.6	32.7	36.4	33.2	32.0	34.8	33.2	33.0	33.5
≥30.0	22.0	16.3	27.4	24.1	18.2	31.5	31.0	25.9	37.2
Leisure-time physical activity (MET hrs/week) median (IQR)	23.1 (30.0)	22.1 (29.4)	23.8 (30.3)	16.3 (23.7)	16.0 (22.9)	16.9 (24.8)	13.7 (22.2)	13.5 (22.0)	14.1 (22.3)
Marital status, %									
Married/with partner	73.2	74.4	72.1	74.2	77.2	70.5	78.9	81.3	75.9
Single	6.4	6.1	6.8	5.2	4.7	5.7	4.8	4.5	5.1
Divorced/separated/widowed	20.3	19.5	21.1	20.6	18.0	23.8	16.4	14.2	19.0
Education, %									
Post-secondary complete	53.7	55.5	51.9	49.2	51.3	46.5	43.8	44.5	42.9
Some post-secondary	21.4	20.2	22.4	21.0	20.7	21.2	22.9	22.4	23.4
High school complete	17.7	17.3	18.1	20.0	19.0	21.3	23.7	23.5	23.9
High school not complete	7.2	6.9	7.5	9.8	8.9	19.3	9.7	9.5	9.9
Annual household income, %									
<$50,000	31.8	31.8	31.7	39.0	37.6	40.9	34.5	33.4	35.9
$50,000–$99,999	38.3	36.9	39.7	37.9	38.9	36.7	40.2	40.0	40.4
≥$100,000	26.9	27.9	26.0	20.0	20.6	19.3	22.6	23.7	21.2
Smoking status, %									
Never smoked	49.7	50.4	49.1	51.4	53.7	48.6	40.8	41.5	39.9
Former smoker	40.6	40.4	40.8	34.7	33.0	36.8	34.5	33.5	35.7
Current smoker	9.6	9.2	10.0	13.8	13.3	14.4	24.7	25.0	24.3
Family history of cancer, %									
No	45.3	47.0	43.7	45.4	45.0	45.9	47.1	47.5	46.8
Yes	54.7	52.9	56.3	54.6	55.0	54.0	52.9	52.6	53.2
Personal history of chronic disease ^a^									
None	57.2	58.1	56.4	57.2	59.4	54.6	60.1	61.6	58.3
One	28.2	28.1	28.3	28.8	27.8	29.9	27.0	25.9	28.2
Two or more	14.5	13.7	15.3	14.0	12.8	15.5	12.9	12.5	13.5
Menopausal status, %									
Pre-menopause	58.9	59.5	58.4	59.4	59.1	59.9	51.8	50.6	53.3
Post-menopause	40.7	40.1	41.4	40.0	40.5	39.3	47.8	49.1	46.2
Hormone replacement therapy use, %									
Never used	84.8	83.3	86.2	82.8	82.9	82.8	86.2	86.9	85.4
Ever used	15.0	16.5	13.6	16.8	16.8	16.9	13.5	12.8	14.4

^a^ Self-reported personal history of one or more of the following: high blood pressure, diabetes, ulcerative colitis, Crohn’s disease, angina, high cholesterol, heart attack, stroke, hepatitis, and cirrhosis of the liver. ^b^ Body mass index was categorized based on Health Canada’s classification scheme [43].

**Table 3:** Largest contributor based on percentage of food groups to daily total energy intake across dietary patterns and different methods to account for misreporting of energy intake.

**Men**							
**Healthy Pattern**							
**Inclusion ^a^ (*n* = 2690)**		**ExBefore ^b^ (*n* = 1780)**		**ExAfter ^c^ (*n* = 1205)**		**InclusionNN ^d^ (*n* = 3468)**	
**Food groups**	**Mean ^e^ (SD)**	**Food groups**	**Mean ^e^ (SD)**	**Food groups**	**Mean ^e^ (SD)**	**Food groups**	**Mean ^e^ (SD)**
Fruit	9.9 (5.4)	Fruit	7.8 (5.0)	Fruit	9.3 (5.2)	Fruit	8.1 (5.4)
Breakfast cereal	4.6 (4.1)	Low-fat dairy	6.0 (6.7)	Fruit juice	4.6 (5.7)	Low-fat dairy	5.9 (6.8)
Fruit juice	4.5 (5.4)	Fruit juice	4.5 (5.6)	Breakfast cereal	4.2 (3.5)	Fruit juice	4.4 (5.4)
Rice	3.6 (6.0)	Breakfast cereal	4.2 (3.4)	Rice	4.0 (6.4)	Breakfast cereal	4.4 (3.8)
Nuts	3.1 (5.0)	Rice	3.3 (5.7)	Nuts	3.7 (5.5)	Rice	3.1 (5.5)
Poultry no skin	3.0 (3.5)	Nuts	3.2 (4.9)	Poultry no skin	3.2 (3.7)	Nuts	2.7 (4.6)
Regular fat dairy	2.7 (3.2)	Poultry no skin	2.9 (3.4)	Regular fat dairy	2.6 (2.9)	Poultry no skin	2.7 (3.3)
Cooked vegetables	1.9 (1.7)	Regular fat dairy	2.1 (2.6)	Cooked vegetables	2.0 (1.8)	Regular fat dairy	2.2 (2.9)
Soup	1.8 (2.1)	Soup	1.7 (1.9)	Soup	1.8 (2.1)	Soup	1.7 (2.0)
Fish	1.6 (1.6)	Cooked vegetables	1.7 (1.6)	Fish	1.6 (1.6)	Cooked vegetables	1.6 (1.5)
Wine	1.5 (3.3)	Fish	1.4 (1.5)	Wine	1.5 (3.5)	Fish	1.4 (1.4)
Legumes	1.2 (1.6)	Wine	1.4 (3.4)	Meal replacement	1.5 (5.3)	Wine	1.4 (3.3)
**Meats/Pizza Pattern**							
**Inclusion ^a^ (*n* =3924)**		**ExBefore ^b^ (*n* = 2127)**		**ExAfter ^c^ (*n* = 2165)**		**InclusionNN ^d^ (*n* = 3760)**	
**Food groups**	**Mean ^e^ (SD)**	**Food groups**	**Mean ^e^ (SD)**	**Food groups**	**Mean ^e^ (SD)**	**Food groups**	**Mean ^e^ (SD)**
Meat	11.6 (5.4)	Meat	10.6 (5.4)	Meat	11.6 (5.4)	Meat	10.3 (5.4)
Pasta/pizza	6.8 (4.7)	Pasta/pizza	6.8 (4.8)	Pasta/pizza	6.9 (4.9)	Pasta/pizza	6.7 (4.6)
Beer	5.6 (11.0)	Beer	5.2 (10.8)	Beer	5.8 (11.1)	Beer	5.0 (11.0)
Regular soda	4.3 (6.4)	Regular soda	5.0 (7.2)	Regular soda	4.5 (6.7)	Regular soda	4.7 (6.9)
Chips	3.6 (3.6)	Chips	3.9 (3.7)	Chips	3.6 (3.5)	Chips	3.8 (3.8)
Other breads	3.5 (3.7)	Processed meat	3.4 (2.6)	Other bread	3.5 (3.8)	Processed meat	3.3 (2.6)
Processed meat	3.5 (2.6)	Regular fat cheese	2.6 (2.8)	Processed meat	3.5 (2.6)	Regular fat cheese	2.4 (2.7)
Regular fat cheese	2.4 (2.8)	French fries	2.2 (2.0)	Regular fat cheese	2.5 (2.8)	French fries	2.1 (2.1)
French fries	2.3 (2.2)	Confectionary	2.2 (3.0)	French fries	2.3 (2.1)	Confectionary	2.1 (2.9)
Eggs	2.2 (2.1)	Liquor	1.9 (5.3)	Eggs	2.0 (1.8)	Liquor	1.9 (5.1)
Liquor	1.9 (5.0)	Regular fat salad dressing	1.5 (1.9)	Liquor	1.9 (5.1)	Regular fat salad dressing	1.5 (1.9)
Regular fat salad dressing	1.5 (2.0)	Mexican	1.2 (1.6)	Regular fat salad dressing	1.5 (1.9)	Mexican	1.3 (1.6)
**Sweets/Dairy Pattern**							
**Inclusion ^a^ (*n* = 3233)**		**ExBefore ^b^ (*n* = 1221)**		**ExAfter ^c^ (*n* = 1758)**		**InclusionNN ^d^ (*n* =2619)**	
**Food groups**	**Mean ^e^ (SD)**	**Food groups**	**Mean ^e^ (SD)**	**Food groups**	**Mean ^e^ (SD)**	**Food groups**	**Mean ^e^ (SD)**
Low fat dairy	7.3 (7.5)	Jam	5.0 (4.7)	Low fat dairy	7.2 (7.3)	Jam	4.5 (4.6)
Wholemeal bread	5.0 (4.9)	Wholemeal bread	4.8 (4.6)	Cake	5.1 (4.6)	Wholemeal bread	4.5 (4.8)
Jam	4.8 (4.5)	Cake	3.9 (4.1)	Wholemeal bread	4.9 (4.5)	Cake	3.5 (3.7)
Cake	4.7 (4.3)	Other bread	3.5 (4.2)	Jam	4.8 (4.5)	Other bread	3.4 (4.1)
Cooked potatoes	3.1 (2.6)	Cooked potatoes	3.2 (2.3)	Cooked potatoes	2.9 (2.3)	Cooked potatoes	3.2 (2.6)
Dessert	2.2 (2.3)	Margarine	2.5 (2.4)	Confectionary	2.3 (3.4)	Margarine	2.1 (2.3)
Confectionary	2.2 (3.2)	Eggs	2.2 (2.0)	Dessert	2.2 (2.3)	Eggs	2.3 (2.3)
Margarine	1.8 (2.1)	Dessert	1.9 (1.9)	Ice cream	1.9 (2.6)	Dessert	1.8 (1.9)
Ice cream	1.8 (2.6)	Coffee	1.8 (0.8)	Margarine	1.9 (2.1)	Coffee	2.1 (1.2)
Coffee	1.3 (1.2)	Ice cream	1.6 (2.4)	Coffee	1.0 (0.9)	Ice cream	1.5 (2.3)
Mayonnaise	0.7 (1.1)	High fat dairy	1.6 (3.9)	Mayonnaise	0.7 (1.1)	High fat dairy	1.4 (3.7)
**Women**							
**Healthy Pattern**							
**Inclusion ^a^ (*n* = 4808)**		**ExBefore ^b^ (*n* = 2919)**		**ExAfter ^c^ (*n* = 2239)**		**InclusionNN ^d^ (*n* = 5633)**	
**Food groups**	**Mean ^e^ (SD)**	**Food groups**	**Mean ^e^ (SD)**	**Food groups**	**Mean ^e^ (SD)**	**Food groups**	**Mean ^e^ (SD)**
Fruit	13.3 (6.3)	Fruit	11.6 (6.0)	Fruit	12.9 (6.0)	Fruit	11.6 (6.5)
Regular fat dairy	5.1 (4.6)	Regular fat dairy	4.4 (3.9)	Regular fat dairy	4.9 (4.1)	Regular fat dairy	4.4 (4.3)
Poultry no skin	4.6 (4.6)	Poultry no skin	4.3 (4.2)	Poultry no skin	4.6 (4.4)	Poultry no skin	4.3 (4.4)
Nuts	3.5 (5.5)	Nuts	4.2 (6.1)	Nuts	4.4 (6.3)	Nuts	3.4 (5.3)
Rice	3.0 (3.7)	Wholemeal bread	3.2 (3.2)	Rice	3.2 (3.9)	Wholemeal bread	3.2 (3.3)
Cooked vegetables	2.6 (2.3)	Rice	3.1 (3.8)	Cooked vegetables	2.6 (2.4)	Rice	3.0 (3.9)
Fish	1.9 (2.2)	Cooked vegetables	2.4 (2.3)	Fish	1.9 (2.1)	Cooked vegetables	2.4 (2.2)
Soup	1.9 (2.2)	Soup	1.9 (2.1)	Soup	1.9 (2.0)	Soup	2.0 (2.3)
Wine	1.7 (3.4)	Fish	1.9 (2.0)	Wine	1.7 (3.6)	Fish	1.9 (2.1)
Legumes	1.5 (1.6)	Wine	1.8 (3.7)	Legumes	1.5 (1.6)	Wine	1.7 (3.6)
Raw vegetables	1.5 (1.1)	Legumes	1.5 (1.5)			Legumes	1.5 (1.6)
Cabbage	1.3 (1.6)	Raw vegetables	1.4 (0.9)			Raw vegetables	1.4 (1.1)
**Meats/Pizza Pattern**							
**Inclusion ^a^ (*n* = 6643)**		**ExBefore ^b^ (*n* = 3835)**		**ExAfter ^c^ (*n* = 3621)**		**InclusionNN ^d^ (*n* = 7049)**	
**Food groups**	**Mean ^e^ (SD)**	**Food groups**	**Mean ^e^ (SD)**	**Food groups**	**Mean ^e^ (SD)**	**Food groups**	**Mean ^e^ (SD)**
Meat	9.2 (4.8)	Meat	8.6 (4.7)	Meat	9.2 (4.7)	Meat	8.4 (4.8)
Pasta/pizza	6.5 (4.4)	Pasta/pizza	6.2 (4.2)	Pasta/pizza	6.4 (4.3)	Pasta/pizza	6.2 (4.3)
Chips	3.8 (4.0)	Chips	3.8 (4.0)	Chips	3.9 (4.1)	Chips	3.7 (4.0)
Regular soda	3.5 (6.6)	Regular soda	3.5 (6.7)	Regular soda	3.6 (6.7)	Regular soda	3.4 (6.6)
Other bread	3.4 (3.6)	Cake	3.3 (3.4)	Other bread	3.3 (3.4)	Cake	3.1 (3.2)
Cooked potatoes	2.8 (2.2)	Other bread	3.1 (3.2)	Cooked potatoes	2.7 (2.0)	Other bread	3.1 (3.4)
Regular fat cheese	2.7 (3.3)	Jam	2.8 (2.9)	Regular fat cheese	2.7 (3.2)	Jam	2.7 (3.0)
Processed meat	2.5 (1.9)	Regular fat cheese	2.7 (3.2)	Confectionary	2.6 (3.9)	Regular fat cheese	2.6 (3.2)
Confectionary	2.5 (3.7)	Cooked potatoes	2.7 (2.0)	Processed meat	2.5 (1.9)	Cooked potatoes	2.8 (2.2)
Eggs	2.2 (2.4)	Confectionary	2.7 (4.0)	Eggs	2.1 (2.1)	Confectionary	2.5 (3.8)
Regular fat salad dressing	2.1 (2.7)	Processed meat	2.4 (1.8)	Regular fat salad dressing	2.1 (2.6)	Processed meat	2.4 (1.9)
Dessert	1.7 (1.9)	Eggs	2.1 (2.1)	Dessert	1.8 (1.9)	Eggs	2.1 (2.3)
**Sweets/Dairy Pattern**							
**Inclusion ^a^ (*n* = 4790)**		**ExBefore ^b^ (*n* = 1873)**		**ExAfter ^c^ (*n* = 2667)**		**InclusionNN ^c^ (*n* = 3559)**	
**Food groups**	**Mean ^e^ (SD)**	**Food groups**	**Mean ^e^ (SD)**	**Food groups**	**Mean ^e^ (SD)**	**Food groups**	**Mean ^e^ (SD)**
Low-fat dairy	10.3 (8.1)	Low-fat dairy	14.3 (6.5)	Low-fat dairy	10.3 (7.6)	Low-fat dairy	13.3 (7.7)
Breakfast cereal	5.1 (4.2)	Breakfast cereal	5.0 (3.7)	Breakfast cereal	4.6 (3.5)	Breakfast cereal	5.2 (4.3)
Wholemeal bread	4.5 (4.3)	Fruit juice	3.8 (4.7)	Wholemeal bread	4.5 (4.0)	Fruit juice	3.7 (4.7)
Fruit juice	4.2 (5.6)			Fruit juice	4.3 (5.5)		
Cake	3.4 (3.4)			Cake	3.7 (3.7)		
Jam	2.9 (2.9)			Jam	3.0 (2.8)		
Ice cream	1.1 (1.9)			Ice cream	1.2 (2.0)		

^a^ Inclusion reports on all participants. Misreporters were included in the k-means cluster analysis. ^b^ ExBefore reports on plausible reporters; however, exclusion of misreporters identified using the revised-Goldberg method was completed before k-means cluster analysis. ^c^ ExAfter reports on plausible reporters; however, exclusion of misreporters identified using the revised-Goldberg method was completed after k-means cluster analysis; ^d^ InclusionNN reports on all participants; however, misreporters identified using the revised-Goldberg method excluded before the cluster analysis but added to the ExBefore cluster solution using the nearest neighbour method; ^e^ Mean percentage contribution by each food group.

**Table 4:** Multivariable Cox proportional hazards ratio of the incidence of All-Cancers for dietary patterns identified by cluster analysis and stratified by four methods to account for misreporting.

**Men**					
**Accounting for Misreporters**	**Dietary Pattern**	***n***	**Cancer Cases ^a^**	**% of Cases Misreport**	**Cancer Risk–HR (95%) ^b^**
Inclusion	Healthy	2690	257	57.2	1.00
	Sweets/Dairy	3233	384	46.6	1.13 (0.96–1.33)
	Meats/Pizza	3924	341	45.6	1.10 (0.93–1.30)
InclusionNN	Healthy	3468	349	47.0	1.00
	Sweets/Dairy	2619	336	47.5	1.11 (0.95–1.30)
	Meats/Pizza	3760	297	52.4	0.95 (0.81–1.11)
ExBefore	Healthy	1780	185		1.00
	Sweets/Dairy	1221	160		1.08 (0.87–1.35)
	Meats/Pizza	2127	156	--	0.85 (0.68–1.06)
ExAfter	Healthy	1205	110		1.00
	Sweets/Dairy	1758	209		1.17 (0.93–1.48)
	Meats/Pizza	2165	182		1.08 (0.84–1.39)
**Women**					
**Accounting for Misreporters**	**Dietary Pattern**	***n***	**Cancer Cases ^a^**	**% of Cases Misreport**	**Cancer Risk–HR (95%) ^c^**
Inclusion	Healthy	4808	347	54.2	1.00
	Sweets/Dairy	4790	419	48.7	1.11 (0.96–1.28)
	Meats/Pizza	6643	528	43.9	1.14 (0.99–1.32)
InclusionNN	Healthy	5633	426	51.9	1.00
	Sweets/Dairy	3559	287	49.0	1.10 (0.94–1.28)
	Meats/Pizza	7049	581	42.9	1.14 (1.00–1.30)
ExBefore	Healthy	2919	205		1.00
	Sweets/Dairy	1873	164		1.28 (1.04–1.58)
	Meats/Pizza	3835	296		1.12 (0.93–1.35)
ExAfter	Healthy	2239	159		1.00
	Sweets/Dairy	2667	235		1.17 (0.96–1.44)
	Meats/Pizza	3621	271		1.12 (0.91–1.38)

^a^ All primary cancer cases except non-melanoma skin cancer. ^b^ Adjusted for age (modelled on a continuous scale), BMI (modelled on a continuous scale), leisure-time physical activity (MET hours/week; modelled on a continuous scale), marital status, educational attainment, smoking status, family history of cancer, and personal history of chronic disease. ^c^ Adjusted for age (modelled on a continuous scale), BMI (modelled on a continuous scale), leisure-time physical activity (MET hours/week; modelled on a continuous scale), marital status, educational attainment, smoking status, family history of cancer, and personal history of chronic disease, menopausal status and hormone replacement therapy usage.

**Table 5:** Multivariable Cox proportional hazards ratio of the incidence of Dietary-Cancers ^a^ stratified by four methods to account for misreporting.

**Men**					
**Accounting for Misreporters**	**Dietary Pattern**	***n***	**Cancer Cases ^a^**	**% of Cases Misreport**	**Cancer Risk-HR** **(95%** **) ^b^**
Inclusion	Healthy	2690	52	63.5	1.00
	Sweets/Dairy	3233	107	50.5	1.34 (0.96–1.89)
	Meats/Pizza	3924	105	38.1	1.42 (1.00–2.02)
InclusionNN	Healthy	3468	73	53.4	1.00
	Sweets/Dairy	2619	110	48.2	1.45 (1.07–1.97)
	Meats/Pizza	3760	81	43.2	1.13 (0.82–1.57)
ExBefore	Healthy	1780	34		1.00
	Sweets/Dairy	1221	57		1.74 (1.12–2.72)
	Meats/Pizza	2127	46		1.23 (0.77–1.95)
ExAfter	Healthy	1205	19		1.00
	Sweets/Dairy	1758	53		1.50 (0.88–2.56)
	Meats/Pizza	2165	65		1.92 (1.12–3.29)
**Women**					
**Accounting for Misreporters**	**Dietary Pattern**	***n***	**Cancer Cases ^a^**	**% of Cases Misreport**	**Cancer Risk-HR (95%) ^c^**
Inclusion	Healthy	4808	241	52.7	1.00
	Sweets/Dairy	4790	284	43.7	1.05 (0.88–1.25)
	Meats/Pizza	6643	380	50.0	1.13 (0.96–1.34)
InclusionNN	Healthy	5633	303	52.1	1.00
	Sweets/Dairy	3559	191	43.5	1.00 (0.84–1.21)
	Meats/Pizza	7049	411	48.7	1.10 (0.94–1.28)
ExBefore	Healthy	2919	145		1.00
	Sweets/Dairy	1873	108		1.17 (0.91–1.50)
	Meats/Pizza	3835	211		1.07 (0.85–1.34)
ExAfter	Healthy	2239	114		1.00
	Sweets/Dairy	2667	160		1.09 (0.86–1.40)
	Meats/Pizza	3621	190		1.02 (0.79–1.30)

^a^ Diet-related cancers based on World Cancer Research Fund/American Institute for Cancer Research Continuous Update Project report. ^b^ Adjusted for age (modelled on a continuous scale), BMI (modelled on a continuous scale), leisure-time physical activity (MET hours/week; modelled on a continuous scale), marital status, educational attainment, smoking status, family history of cancer, and personal history of chronic disease. ^c^ Adjusted for age (modelled on a continuous scale), BMI (modelled on a continuous scale), leisure-time physical activity (MET hours/week; modelled on a continuous scale), marital status, educational attainment, smoking status, family history of cancer, and personal history of chronic disease, menopausal status and hormone replacement therapy usage.

**Table 6:** Multivariable cox proportional hazards ratio of the incidence of Digestive-Cancers ^a^ stratified by four methods to account for misreporting.

**Men**					
**Accounting for Misreporters**	**Dietary Pattern**	***n***	**Cancer Cases ^a^**	**% of Cases Misreport**	**Cancer Risk-HR (95%) ^b^**
Inclusion	Healthy	2690	38	57.9	1.00
	Sweets/Dairy	3233	76	51.3	1.43 (0.96–2.13)
	Meats/Pizza	3924	77	40.3	1.45 (0.96–2.17)
InclusionNN	Healthy	3468	58	44.8	1.00
	Sweets/Dairy	2619	69	56.5	1.23 (0.86–1.76)
	Meats/Pizza	3760	64	42.2	1.10 (0.77–1.60)
ExBefore	Healthy	1780	32		1.00
	Sweets/Dairy	1221	30		1.08 (0.64–1.82)
	Meats/Pizza	2127	37		1.01 (0.61–1.67)
ExAfter	Healthy	1205	16		1.00
	Sweets/Dairy	1758	37		1.37 (0.76–2.49)
	Meats/Pizza	2165	46		1.62 (0.89–2.95)
**Women**					
**Accounting for Misreporters**	**Dietary Pattern**	***n***	**Cancer Cases ^a^**	**% of Cases Misreport**	**Cancer Risk-HR (95%) ^c^**
Inclusion	Healthy	4808	51	52.9	1.00
	Sweets/Dairy	4790	69	34.8	1.17 (0.81–1.69)
	Meats/Pizza	6643	81	50.6	1.22 (0.84–1.77)
InclusionNN	Healthy	5633	60	51.7	1.00
	Sweets/Dairy	3559	46	34.8	1.25 (0.84–1.84)
	Meats/Pizza	7049	98	49.0	1.43 (1.02–2.01)
ExBefore	Healthy	2919	29		1.00
	Sweets/Dairy	1873	30		1.73 (1.03–2.89)
	Meats/Pizza	3835	50		1.43 (0.88–2.33)
ExAfter	Healthy	2239	24		1.00
	Sweets/Dairy	2667	45		1.42 (0.86–2.35)
	Meats/Pizza	3621	40		1.13 (0.66–1.93)

^a^ Digestive system cancers based on World Health Organization classification. ^b^ Adjusted for age (modelled on a continuous scale), BMI (modelled on a continuous scale), leisure-time physical activity (MET hours/week; modelled on a continuous scale), marital status, educational attainment, smoking status, family history of cancer, and personal history of chronic disease. ^c^ Adjusted for age (modelled on a continuous scale), BMI (modelled on a continuous scale), leisure-time physical activity (MET hours/week; modelled on a continuous scale), marital status, educational attainment, smoking status, family history of cancer, and personal history of chronic disease, menopausal status and hormone replacement therapy usage.

## Supplementary Materials

The following are available online at https://www.mdpi.com/2072-6643/11/11/2614/s1. Tables S1–S3: Competing risk hazard ratios for All-Cancers, Dietary-Cancers, and Digestive-Cancers.
