# Supplementary material for: Strategies to Address Misestimation of Energy Intake Based on Self-Report Dietary Consumption in Examining Associations Between Dietary Patterns and Cancer Risk
Source: Nutrients. 2019 Nov 1;11(11):2614. doi: 10.3390/nu11112614 (PMC6893710; doi:10.3390/nu11112614)
Supplement: Supplementary file 1 [file nutrients-11-02614-s001.pdf]

nutrients

Table S1 – Multivariable Cox proportional hazards ratio, from competing risk analysis to account for deaths before ACR linkage date in participants who were cancer-free during follow-up, of the incidence of All-Cancers for dietary patterns identified by cluster analysis and stratified by four methods to account for misreporting

		Men				Women			
**Accounting for misreporters**	**Dietary pattern**	**N**	**Cancer cases[a]**	**% of cases misreport**	**Cancer risk – HR [95%]**	**N**	**Cancer cases[a]**	**% of cases misreport**	**Cancer risk – HR [95%]**
Inclusion	Healthy	2690	257	57.2	1.00	4808	347	54.2	1.00
	Sweets/Dairy	3233	384	46.6	1.13 [0.96 - 1.33]	4790	419	48.7	1.11 [0.96 - 1.28]
	Meats/Pizza	3924	341	45.6	1.10 [0.93 - 1.31]	6643	528	43.9	1.14 [0.99 - 1.32]
InclusionNN	Healthy	3468	349	47.0	1.00	5633	426	51.9	1.00
	Sweets/Dairy	2619	336	47.5	1.11 [0.95 - 1.30]	3559	287	49.0	1.10 [0.94 - 1.28]
	Meats/Pizza	3760	297	52.4	0.95 [0.81 - 1.11]	7049	581	42.9	**1.14 [1.00 - 1.30]**
ExBefore	Healthy	1780	185	--	1.00	2919	205	--	1.00
	Sweets/Dairy	1221	160	--	1.08 [0.87 - 1.35]	1873	164	--	**1.28 [1.04 - 1.58]**
	Meats/Pizza	2127	156	--	0.85 [0.68 - 1.06]	3835	296	--	1.12 [0.93 - 1.35]
ExAfter	Healthy	1205	110	--	1.00	2239	159	--	1.00
	Sweets/Dairy	1758	209	--	1.17 [0.93 - 1.48]	2667	235	--	1.17 [0.96 - 1.44]
	Meats/Pizza	2165	182	--	1.08 [0.84 - 1.39]	3621	271	--	1.12 [0.91 - 1.38]

a – All primary cancer cases except non-melanoma skin cancer

Table S2 - Multivariable Cox proportional hazards ratio, from competing risk analysis to account for deaths before ACR linkage date in participants who were cancer-free during follow-up, of the incidence of Dietary-Cancers[a] stratified by four methods to account for misreporting.

		Men				Women			
**Accounting for misreporters**	**Dietary pattern**	**N**	**Cancer cases[a]**	**% of cases misreport**	**Cancer risk - HR [95%]**	**N**	**Cancer cases[a]**	**% of cases misreport**	**Cancer risk - HR [95%]**
Inclusion	Healthy	2690	52	63.5	1.00	4808	241	52.7	1.00
	Sweets/Dairy	3233	107	50.5	1.35 [0.96 - 1.89]	4790	284	43.7	1.05 [0.88 - 1.25]
	Meats/Pizza	3924	105	38.1	**1.42 [1.00 - 2.02]**	6643	380	50.0	1.13 [0.96 - 1.34]
InclusionNN	Healthy	3468	73	53.4	1.00	5633	303	52.1	1.00
	Sweets/Dairy	2619	110	48.2	**1.45 [1.07 - 1.97]**	3559	191	43.5	1.00 [0.84 - 1.21]
	Meats/Pizza	3760	81	43.2	1.13 [0.82 - 1.57]	7049	411	48.7	1.10 [0.94 - 1.28]
ExBefore	Healthy	1780	34	--	1.00	2919	145	--	1.00
	Sweets/Dairy	1221	57	--	**1.74 [1.12 - 2.72]**	1873	108	--	1.17 [0.91 - 1.50]
	Meats/Pizza	2127	46	--	1.23 [0.77 - 1.95]	3835	211	--	1.07 [0.85 - 1.34]
ExAfter	Healthy	1205	19	--	1.00	2239	114	--	1.00
	Sweets/Dairy	1758	53	--	1.50 [0.88 - 2.56]	2667	160	--	1.09 [0.86 - 1.40]
	Meats/Pizza	2165	65	--	**1.92 [1.12 - 3.29]**	3621	190	--	1.02 [0.79 - 1.30]

a – Diet-related cancers based on World Cancer Research Fund/American Institute for Cancer Research Continuous Update Project (WCRF/AICR CUP) report

Table S3 - Multivariable cox proportional hazards ratio, from competing risk analysis to account for deaths before ACR linkage date in participants who were cancer-free during follow-up, of the incidence of Digestive-Cancers[a] stratified by four methods to account for misreporting

		Men				Women			
**Accounting for misreporters**	**Dietary pattern**	**N**	**Cancer cases[a]**	**% of cases misreport**	**Cancer risk - HR [95%]**	**N**	**Cancer cases[a]**	**% of cases misreport**	**Cancer risk - HR [95%]**
Inclusion	Healthy	2690	38	57.9	1.00	4808	51	52.9	1.00
	Sweets/Dairy	3233	76	51.3	1.43 [0.96 - 2.13]	4790	69	34.8	1.17 [0.81 - 1.69]
	Meats/Pizza	3924	77	40.3	1.45 [0.96 - 2.18]	6643	81	50.6	1.22 [0.84 - 1.77]
InclusionNN	Healthy	3468	58	44.8	1.00	5633	60	51.7	1.00
	Sweets/Dairy	2619	69	56.5	1.23 [0.86 - 1.76]	3559	46	34.8	1.25 [0.84 - 1.84]
	Meats/Pizza	3760	64	42.2	1.10 [0.77 - 1.60]	7049	98	49.0	**1.43 [1.02 - 2.01]**
ExBefore	Healthy	1780	32	--	1.00	2919	29	--	1.00
	Sweets/Dairy	1221	30	--	1.08 [0.64 - 1.82]	1873	30	--	**1.73 [1.03 - 2.89]**
	Meats/Pizza	2127	37	--	1.01 [0.61 - 1.67]	3835	50	--	1.43 [0.88 - 2.33]
ExAfter	Healthy	1205	16	--	1.00	2239	24	--	1.00
	Sweets/Dairy	1758	37	--	1.37 [0.76 - 2.49]	2667	45	--	1.42 [0.86 - 2.35]
	Meats/Pizza	2165	46	--	1.62 [0.89 - 2.95]	3621	40	--	1.13 [0.66 - 1.93]

a – Digestive system cancers based on World Health Organization (WHO) classification
